# Comparative transcriptome profiling of immune response against *Vibrio harveyi* infection in Chinese tongue sole

**DOI:** 10.1038/s41597-019-0231-2

**Published:** 2019-10-22

**Authors:** Hao Xu, Xiwen Xu, Xihong Li, Lei Wang, Jiayu Cheng, Qian Zhou, Songlin Chen

**Affiliations:** 1 Yellow Sea Fisheries Research Institute, Chinese Academy of Fishery Sciences, Key Laboratory of Sustainable Development of Marine Fisheries, Ministry of Agriculture; Laboratory for Marine Fisheries Science and Food Production Processes, Pilot National Laboratory for Marine Science and Technology (Qingdao), Qingdao, China; 2 College of Fisheries and Life Science, Shanghai Ocean University, Shanghai, China; 3 Tangshan Center of Engineering and Technology for flatfish Aquaculture, Weizhuo Aquaculture Co. Ltd, Tangshan, China

**Keywords:** Infection, Gene expression

## Abstract

*Vibrio harveyi* is a major bacterial pathogen that causes fatal vibriosis in Chinese tongue sole (*Cynoglossus semilaevis*), resulting in massive mortality in the farming industry. However, the molecular mechanisms of *C*. *semilaevis* response to *V*. *harveyi* infection are poorly understood. Here, we performed transcriptomic analysis of *C*. *semilaevis*, comparing resistant and susceptible families in response to *V*. *harveyi* challenge (CsRC and CsSC) and control conditions (CsRU and CsSU). RNA libraries were constructed using 12 RNA samples isolated from three biological replicates of the four groups. We performed transcriptome sequencing on an Illumina HiSeq platform, and generating a total of 1,095 million paired-end reads, with the number of clean reads per library ranging from 75.27 M to 99.97 M. Through pairwise comparisons among the four groups, we identified 713 genes exhibiting significant differences at the transcript level. Furthermore, the expression levels were validated by real-time qPCR. Our results provide a valuable resource and new insights into the immune response to *V*. *harveyi* infection.

## Abstract (summary)

**Table:** 

Measurement(s)	messenger RNA
Technology Type(s)	RNA sequencing
Factor Type(s)	experimental condition • Phenotypic variability
Sample Characteristic - Organism	Cynoglossus semilaevis
Sample Characteristic - Environment	aquatic natural environment

Machine-accessible metadata file describing the reported data: 10.6084/m9.figshare.9907706

## Background & Summary

Knowledge of fish immune systems contributes to understanding the evolution of the immune system, and there is an increasing interest in fish immunology for its unique position in the evolutionary spectrum from lower vertebrates to higher vertebrates^1^. Meanwhile, infectious pathogens, such as bacteria, mould, viruses and protozoans, cause a mass mortality in commercial fish, therefore, it is urgent to study the underlying molecular mechanisms of fish immunology, and to explore novel methods to enhance defences against pathogens in fish^2,3^.

Previous studies on immune analyses in fish have primarily concentrated on several important genes in model species^4,5^, while the response against bacterial infection in other immune-regulated genes is still unclear. Nevertheless, transcriptomic profiling using next-generation sequencing technologies provides a new approach to studying fish immunology in various marine aquatic species. For example, transcriptomic profiling is conducted to evaluate whole-genome expression patterns in the immune response against bacterial and viral infection to analyze any relevant differences observed. In *Epinephelus coioides*, transcriptome analysis during *Vibrio alginolyticus* infection revealed changes in immune gene expression with concomitant induction of innate immune-related complement and hepcidin systems^6^. Transcriptomic analysis of *Salmo salar* harbouring an infectious salmon anemia virus revealed 3,023 differentially expressed transcripts, with extreme differences in the expression of viral segments between susceptible and resistant groups^7^. Furthermore, transcriptomic profiling sheds lights on potentially novel immune-related transcripts. Transcriptome analysis of *C*. *semilaevis* responding to *Vibrio anguillarum* infection identified multiple differentially expressed annotated and novel genes, which were mostly relevant to the immune response, immune system regulation, and cytokine production^8^. Taken together, these transcriptomic analyses of the response to bacterial and viral infection in teleosts allow us to understand the molecular mechanisms of immune response and to identify novel genes associated with bacterial infection.

*C*. *semilaevis* is a valuable marine aquatic species distributed in Northern China^9^. However, vibriosis, which is caused by various bacteria such as *Vibrio harveyi*, *Vibrio anguillarum*, *Vibrio alginolyticus*, *Vibrio Parahemolyticus*, *Vibrio rotiferianus*, and *Vibrio aestuarianus*, has severely disrupted the development of *C*. *semilaevis* aquaculture. In *C*. *semilaevis* farming, *V*. *harveyi* is a major pathogen, causing severe infectious vibriosis with symptoms of putrefied skin, ascites, and tail rot. Although some studies examining *C*. *semilaevis* with *V*. *harveyi* infection have been reported^10,11^, the underlying molecular mechanisms mounted against *V*. *harveyi* infection by the host have not been extensively studied, and the exploitation of genetic resources is required. To address this knowledge gap, we selected two *C*. *semilaevis* families based on their significant mortality differences after *V*. *harveyi* infection. One family with a high mortality rate (cumulative mortality rate, CMR, >80%) was considered the *V*. *harveyi* susceptible family, whereas the other one with a low mortality rate (CMR < 20%) was considered the *V*. *harveyi* resistant family. Understanding the different immune molecular mechanisms will be very helpful for enhancing host ability against *V*. *harveyi* infection and for breeding *V*. *harveyi* resistant strains of *C*. *semilaevis*.

Herein, we performed the transcriptome analyses of two phenotypes of *C*. *semilaevis* (susceptible and resistant to *V*. *harveyi*) under *V*. *harveyi* challenge and control conditions. We discribe the detailed procedure of our experimental design including the treatment of fish, tissues collection, library construction and transcriptome sequencing. Quality control was conducted to evaluate the quality of our transcriptome data using FastQC, and a high-quality dataset is presented. Additionally, we performed comparative transcriptomic analyses of four *C*. *semilaevis* groups with the aim of screening key genes that cause the differences in disease resistance between resistant and susceptible families and providing an improved understanding of the immune response to *V*. *harveyi* infection.

## Methods

### Ethical approval

The collection and handling of the animals in the study was approved by the Animal Care and Use Committee of Chinese Academy of Fishery Sciences’, and all animals and experiments were conducted in accordance with the guidelines for the care and use of laboratory animals at the Chinese Academy of Fishery Sciences.

### Fish rearing and bacterial challenge

The fish (109 ± 24.8 g) used in this experiment were obtained from two *C*. *semilaevis* families described above at the Haiyang High-Tech Experimental Base (Shandong, China). Fish were kept in seawater ponds with a continuous supply of seawater at a temperature of 20~23 °C. After 7 days’ acclimation, the fish were challenged with *Vibrio harveyi* (kept by Key Laboratory for Sustainable Utilization of Marine Fisheries Resources). A pre-test was conducted to confirm the concentration of *V*. *harveyi* (8*10^4^ cfu/ml). Fish were randomly selected from the two families and challenged with the same concentration of *V*. *harveyi* by intraperitoneal injection based on their weights (2 ml/kg). Fish were sampled before injection and 24 h after infection, and the liver, spleen, and kidney tissues were collected from three individual fish in each group and immediately frozen in liquid nitrogen. Tissues were stored at −80 °C until RNA extraction. All fish were anesthetized with a lethal dose of MS-222 (300 ppm) to prevent suffering. The unchallenged and challenged resistant families of *C*. *semilaevis* were termed the CsRU and CsRC groups, respectively. The unchallenged and challenged susceptible family of *C*. *semilaevis* were termed the CsSU and CsSC groups, respectively. Three samples were used in each group (Table 1).

**Table 1:** Accession numbers for each biological sample.

Organism	analysis type	Sample name	Replicate	Group	Accession number (Sample)
*Cynoglossus semilaevis*	RNA-sequencing	SU1	Biological Replicate 1	CsSU	GSM3619558
*Cynoglossus semilaevis*	RNA-sequencing	SU2	Biological Replicate 2	CsSU	GSM3619559
*Cynoglossus semilaevis*	RNA-sequencing	SU3	Biological Replicate 3	CsSU	GSM3619560
*Cynoglossus semilaevis*	RNA-sequencing	RU1	Biological Replicate 1	CsRU	GSM3619561
*Cynoglossus semilaevis*	RNA-sequencing	RU2	Biological Replicate 2	CsRU	GSM3619562
*Cynoglossus semilaevis*	RNA-sequencing	RU3	Biological Replicate 3	CsRU	GSM3619563
*Cynoglossus semilaevis*	RNA-sequencing	SC1	Biological Replicate 1	CsSC	GSM3619564
*Cynoglossus semilaevis*	RNA-sequencing	SC2	Biological Replicate 2	CsSC	GSM3619565
*Cynoglossus semilaevis*	RNA-sequencing	SC3	Biological Replicate 3	CsSC	GSM3619566
*Cynoglossus semilaevis*	RNA-sequencing	RC1	Biological Replicate 1	CsRC	GSM3619567
*Cynoglossus semilaevis*	RNA-sequencing	RC2	Biological Replicate 2	CsRC	GSM3619568
*Cynoglossus semilaevis*	RNA-sequencing	RC3	Biological Replicate 3	CsRC	GSM3619569

### RNA extraction, library construction, RNA sequencing

Total RNA was extracted with TRIzol reagents (Invitrogen, USA) following the instructions of the manufacturer. Purified RNA was quantified using Qubit® RNA Assay Kit in a Qubit® 2.0 Fluorimeter (Life Technologies, CA, USA), and its integrity was evaluated using the RNA Nano 6000 Assay Kit and the Bioanalyzer 2100 system (Agilent Technologies, CA, USA). Equal amounts of total RNA from the kidney, spleen, and liver of individual fish were pooled to generate the RNA sample preparation as one biological replicate. Three biological replicates of each group were used to construct cDNA libraries following the Illumina standard operating procedure. Libraries were sequenced on an Illumina HiSeq platform to generate 150 bp paired-end reads.

### Quality validation, data cleaning and normalization

We used FastQC^12^ to assess the quality of raw reads in fastq format, and all results were merged and visualized using MultiQC^13^. Clean reads were generated from raw reads by removing low quality reads and those containing adapters, poly-N using RNA-QC-Chain^14^ with default parameters, then mapped onto the *C*. *semilaevis* reference genome (Accession no. GCF_000523025.1) using TopHat software with the parameter of mismatch = 2. We then used Cufflinks with default parameters to construct and identify both known and novel transcripts from TopHat alignment results^15^. Subsequently, we used HTSeq.^16^ to count the number of fragments mapped to each gene with the parameters: -m union, -s no, and the expected number of fragments per kilobase of transcript sequence per Millions base pairs (FPKM) were calculated to assess the expression levels.

### Downstream analysis

**Fig. 1:**
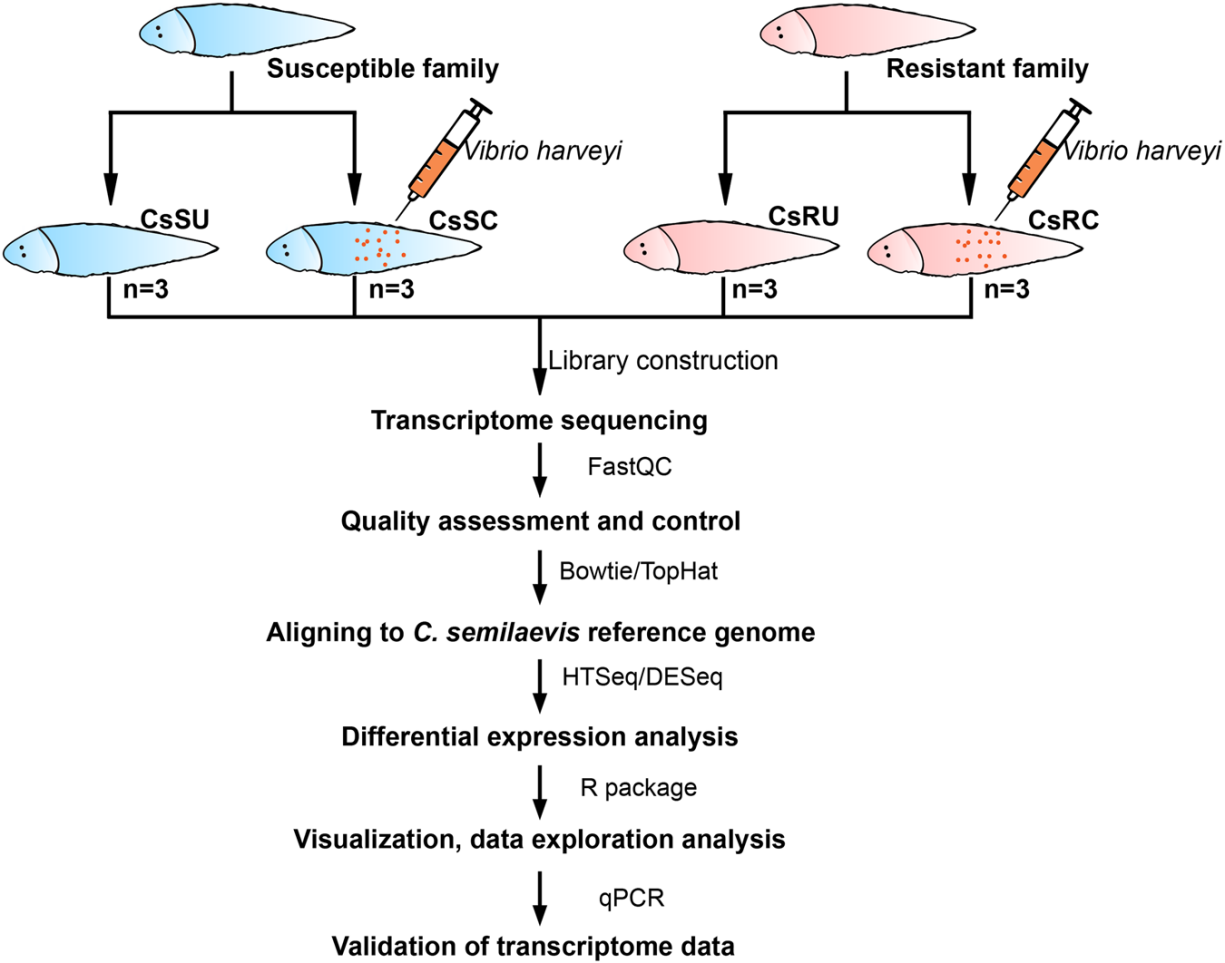
Overview of the experimental design. The flowchart represents RNA-Seq workflow and bioinformatics analysis workflow.

We used the DESeq package to conduct differential expression analysis^17^ and the *p*-values were adjusted by the Benjamini & Hochberg method for controlling the false discovery rate^18^. Genes with an adjusted *p*-value < 0.05 were considered differentially expressed genes (DEGs). Furthermore, we calculated the Pearson correlation between samples according to gene expression profiles and the correlation matrix was visualized using ggplot2^19^. Box plots, volcano plots, heat maps and Venn diagrams were drawn using R packages. The analysis workflow is shown in Fig. 1.

### Real-time qPCR validation

In this study, we randomly selected 24 genes for real-time qPCR validation to confirm the results of differential expression analysis. Real-time qRCR was performed with SYBR^®^ Premix Ex Taq™ (TaKaRa, Japan) on an ABI 7500 Fast Real-Time PCR system (Applied Biosystems, USA) under the following conditions: denaturation at 95 °C for 30 s, then 40 cycles of 95 °C for 15 s, 60 °C for 20 s, and 72 °C for 10 s. Relative gene expression was analyzed by 2^−ΔΔCt^ method. β-actin was chosen as the internal control for normalization^20^. We used Prism software to determine statistical significance and draw plots.

## Data Records

Raw FASTQ files were deposited into the NCBI Sequence Read Archive (SRA) with accession number SRP186770 (Table 1)^21^. The abundance count for all the samples was deposited to the NCBI Gene Expression Omnibus (GEO) with accession number GSE126995^22^. The DEGs presented in the Venn diagram are available on Figshare^23^.

## Technical Validation

**Fig. 2:**
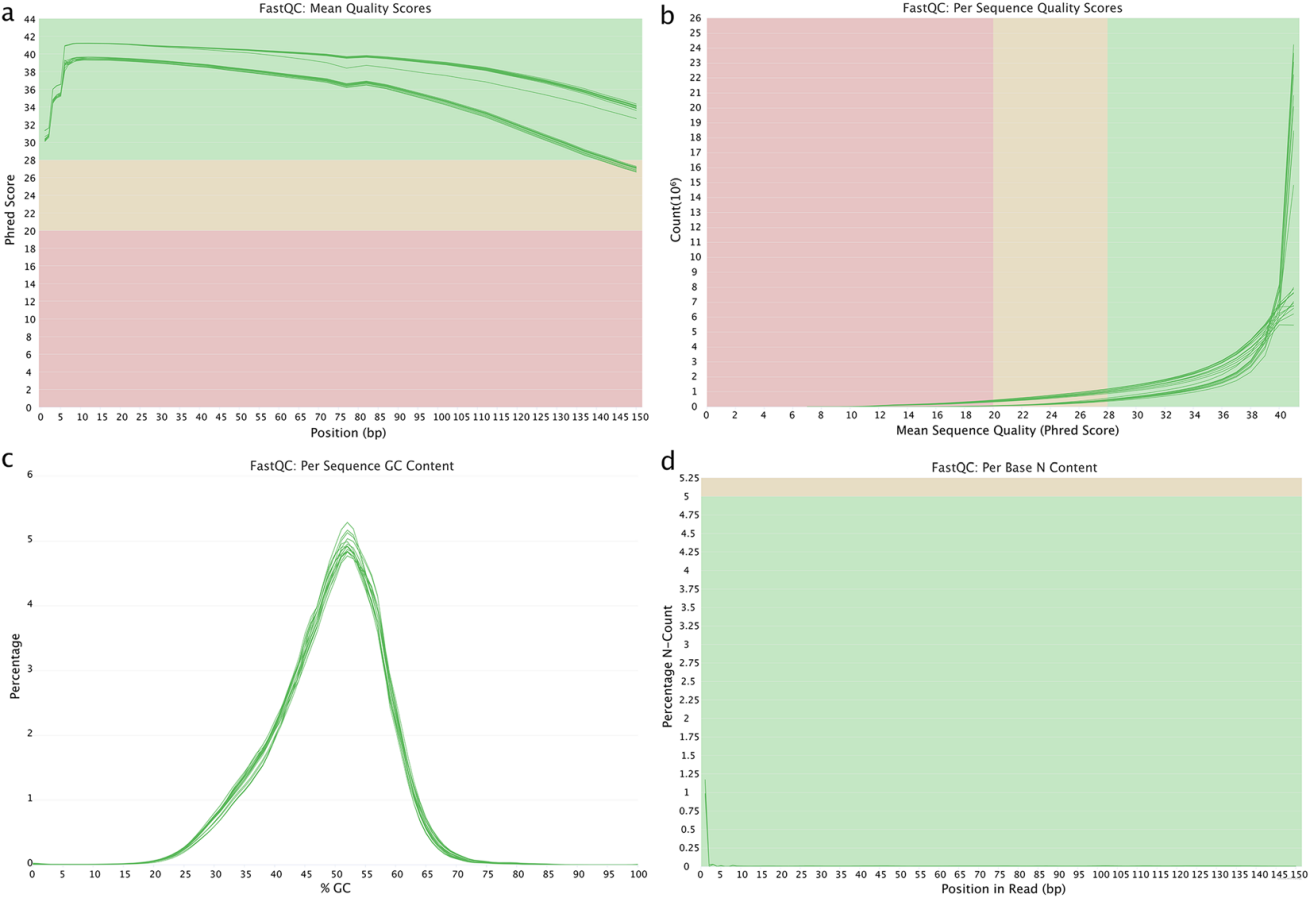
Visualization of qualities of *C*. *semilaevis* sequencing data. (**a**) Per base sequence quality. (**b**) Per sequence quality scores. (**c**) Per sequence GC content. (**d**) Per base N content.

All RNA samples used for library construction had 260:280 ratios of ≥1.5 and an RNA integrity number (RIN) of ≥8. We constructed 12 RNA libraries of mixed tissues with three biological replicates from four groups (CsSU, CsRU, CsSC, and CsRC) (Fig. 1). We applied FastQC and RNA-QC-Chain to verify that the data was suitable for downstream analysis (Fig. 2, Table 2).

**Table 2:** Summary statistics for the sequencing data of the twelve samples.

Sample name	Number of raw reads	Number of clean reads	clean bases	Error rate(%)	Q20(%)	Q30(%)	GC content(%)
SU1	93,383,974	87,594,930	13.14 G	0.02	94.3	88.31	48.74
SU2	104,672,142	98,188,152	14.73 G	0.03	94.34	88.28	49.11
SU3	80,095,718	75,276,260	11.29 G	0.02	94.37	88.36	48.61
RU1	85,660,884	80,441,096	12.07 G	0.02	94.25	88.16	48.7
RU2	91,134,342	85,816,620	12.87 G	0.02	94.36	88.31	48.98
RU3	91,226,452	85,555,254	12.83 G	0.03	93.73	87.06	48.7
SC1	101,900,430	95,811,584	14.37 G	0.02	94.36	88.39	48.22
SC2	104,216,082	97,740,946	14.66 G	0.03	94.18	88.03	48.19
SC3	100,320,038	93,866,088	14.08 G	0.03	94.02	87.83	47.66
RC1	106,581,728	99,971,142	15 G	0.02	94.3	88.31	48.6
RC2	105,878,506	99,234,478	14.89 G	0.03	94.27	88.26	48.31
RC3	100,828,908	95,019,216	14.25 G	0.02	94.52	88.63	48.24

**Fig. 3:**
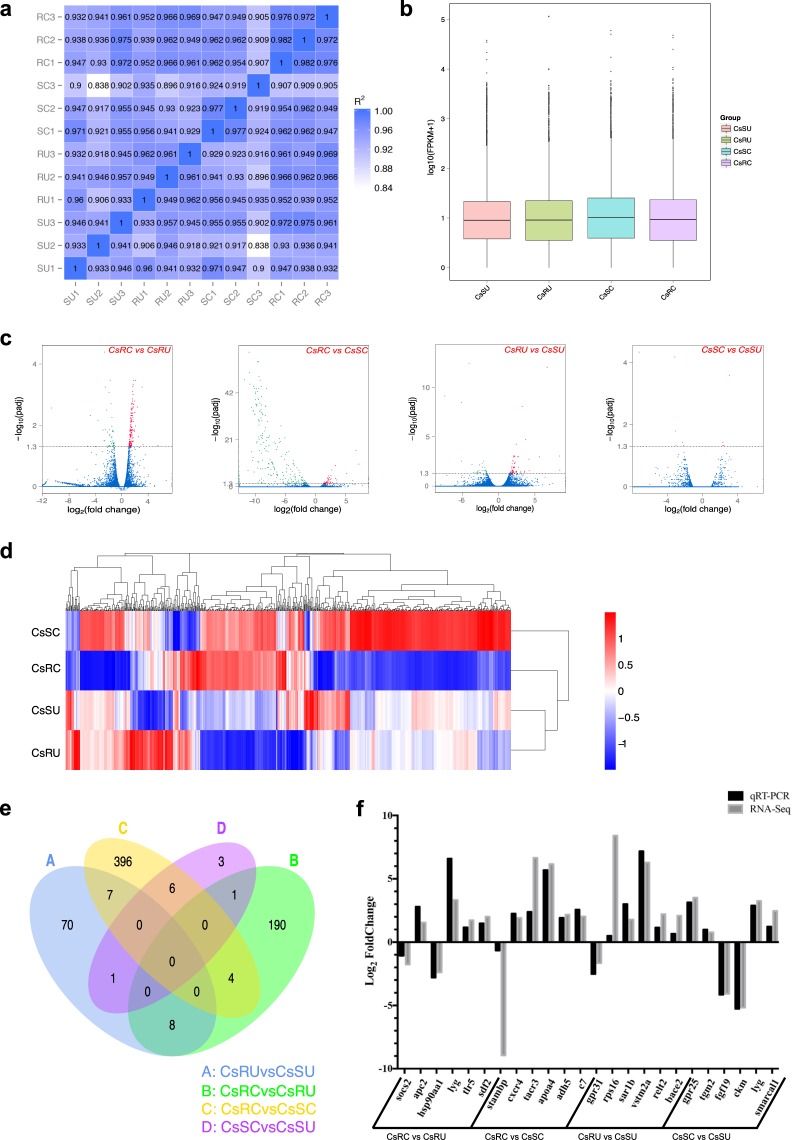
Quality assessment and comparisons of transcriptome data among the *C*. *semilaevis* groups. (**a**) Correlation matrix of the transcriptome data of all the samples. (**b**) Boxplot of FPKM distribution among the four groups. (**c**) Volcanoplot of differentially expressed genes (DEGs) distribution in the four pairwise comparisons. (**d**) Hierarchical cluster analysis of gene expression profiles of the four groups. (**e**) Venn diagram of the number of shared DEGs between contrasts. (**f**) Validation of differential expression of 24 genes from qPCR and RNA-Seq.

After clean reads were mapped onto the *C*. *semilaevis* reference genome, we calculated the number and percentage of uniquely mapped reads and multiply mapped reads in Table 3. The correlation of gene expression levels between samples is an important index to verify the reliability of an experiment, and the square of the Pearson correlation coefficient (R2) of >0.9 was a prerequisite for differential expression analysis (Fig. 3a). The FPKM boxplot shows the distribution of gene expression levels in Fig. 3b. Additionally, we analyzed the expression profiles among the four groups in the pairwise comparisons. As shown in Fig. 3c, downregulated and upregulated DEGs are highlighted in green and red with a threshold of −log_10_ (adjusted *p*-value) ≥1.3, respectively. Furthermore, a cluster analysis of the DEGs indicated that the expression patterns of those groups differed significantly from each other (Fig. 3d). We identified a total of 713 DEGs in four pairwise comparisons (CsRC vs CsRU, CsRC vs CsSC, CsRUvs CsSU and CsSC vs CsSU) (Fig. 3e). Although the values of the log_2_ fold change from the transcriptomic analysis and qPCR analysis were different, the differential expression levels of these selected genes by qPCR were highly consistent with those observed by RNA-Seq (Fig. 3f). The primers for these genes are shown in Table 4.

**Table 3:** Statistics analysis of clean reads mapping onto reference genome.

Sample name	Number of uniquely mapped reads	Percentage of uniquely mapped reads %	Number of multiply mapped reads	Percentage of multiply mapped reads %
SU1	60,754,188	69.36	1,605,970	1.83
SU2	68,745,699	70.01	2,265,076	2.31
SU3	52,489,583	69.73	1,378,661	1.83
RU1	56,281,304	69.97	1,259,706	1.57
RU2	60,241,555	70.2	1,469,368	1.71
RU3	59,485,111	69.53	1,256,973	1.47
SC1	66,388,773	69.29	1,753,415	1.83
SC2	67,065,736	68.62	2,025,623	2.07
SC3	64,134,171	68.33	1,305,320	1.39
RC1	69,465,962	69.49	1,793,586	1.79
RC2	68,779,401	69.31	1,908,332	1.92
RC3	67,008,296	70.52	1,791,000	1.88

**Table 4:** Primers of selected genes for qPCR validation.

Gene	Forward Primer	Reverse Primer
*socs2*	TTCAAACTGGACTCGGTGGTTCT	CAGTTGTTGGTGGTGCTGCTAAT
*apc2*	TCGACGATGAGGCAAAGAGGATT	TTTCTTTGGTTTGCCACCCTGTC
*hsp90aa1*	TAAGCTGTATGTGCGCAGAGTCT	TTGCGGATGACCTTCAGGATCTT
*lyg*	TGCCAGAGGTGAATGGAATAGCA	AGTAGTCTCCCCCTGTCGTGTAT
*tlr5*	ATCTCCCTGATCCTGACAACAGC	AATTGATCCTGCAGACCCTCGAA
*sdf2*	TTCTGAGTGTGACAGGGGAACAG	GGCTGTATGAAGACACCCTCCAT
*stambp*	TGGCAAATTGACCAGAAATGCGT	TGTGGGGTGGGTATGTATCCAAC
*cxcr4*	GATCCAAATGCAGCCTTACGGAC	CTAGGATGAGGACACTGCCGTAC
*tacr3*	GGGAGGCTTACTGCAAATTCCAC	CAAACGATAACTCCTGTGGTGGC
*apoa4*	CCTCATCTCTCAGAGCACCAAGG	AGTTCTGACATCATCTCCTCGGC
*adh5*	AATGCACAAAGATGGCTTCCCAG	GGGAGACGAACAGAGGAATCACA
*c7*	ACGCAGCCTACAGGAAGGTTATT	GTACGCTCTTGATGGTCCAGAGT
*gpr31*	TGGCCATATACAACAGCACCAGA	GATGGGTAAAAGGGCTGCATGTC
*rps16*	GGGGAATGGTCTGATCAAGGTGA	CCTGACGGATGGCATAGATCTGT
*sar1b*	CTGGCTGAGGCTAAGACTGAACT	CCAAACATGCACCTGAGACCATC
*vstm2a*	GGAGATGGAGATGATACCGGAGC	ACCCTGCATTCGTAGAGACCTTC
*relt2*	AGGTTTCGTAAGGAGTCCATCGG	AATCTTCCCACAGAGAACACCGT
*bace2*	TCCGTATCACCATTCTGCCTCAG	CCAGTCTCTTCTGCACTCGATCA
*gpr25*	GACGCAGACACTCCCTCAAAATG	CCAGACAACAGGAGATGACCAGT
*tgm2*	ACCAAAACAAGCTGCACCATCAA	ATCCACAGTTCCCTCCCAGATTG
*fgf19*	GATCCAGGTTGTGTTGCCATCAG	TTTGTCGGAGGTGTAGACGTTGT
*ckm*	CACACGCCAAGTTTGAGGAGATC	CCATCAGCTTGACACCATCAACC
*lyg*	AGGATATGGCGATGGAGGGAATG	AAGATCTCAGTGCCTTGCTCGAT
*smarcal1*	ATGTTGTCAAGGTTTGCCAGTGG	GTCCTCTCCTCCATCACTTTCCC

Taken together, our findings present a high-quality transcriptomic dataset characterizing the *C*. *semilaevis* response to *V*. *harveyi* infection. Additionally, we screened multiple genes associated with the immune response to *V*. *harveyi* infection. The dataset provides a valuable resource for isolating the immune-related genes, for better understanding the biological process of disease resistance, and for exploring reliable ways of host immune defence against *V*. *harveyi*.

## Data Availability

The softwares used for data processing are included in the methods and available in the following list: 1. FastQC v0.11.6 was used for quality assessment of FASTQ data: http://www.bioinformatics.babraham.ac.uk/projects/fastqc/. 2. MultiQC was used for combining fastqc results into one: https://pypi.python.org/pypi/multiqc. 3. RNA-QC-Chain was used for data preprocessing of raw data: http://bioinfo.single-cell.cn/rna-qc-chain.html. 4. TopHat v2.0.12 was used for clean reads aligned to the reference genome: http://ccb.jhu.edu/software/tophat/downloads/. 5. Cufflinks v2.1.1 was used for transcript assembly of samples: http://cole-trapnell-lab.github.io/cufflinks/. 6. HTSeq v0.6.1 was used for counting the reads numbers mapped to each gene: https://htseq.readthedocs.io/en/release_0.11.1/history.html#version-0-6-1. 7. DESeq package v1.18.0 was used for differential expression analysis of two groups with biological replicates: https://bioconductor.riken.jp/packages/3.0/bioc/html/DESeq.html. 8. Ggplot2 package was used for visualization of a correlation matrix between samples: http://www.sthda.com/english/wiki/ggcorrplot-visualization-of-a-correlation-matrix-using-ggplot2.
